# The Great Northern Central Hospital

**Published:** 1895-03-02

**Authors:** 


### THE PAY SYSTEM.

### THE GREAT NORTHERN CENTRAL HOSPITAL.

#### First Annual Meeting in the New Hospital.

The annual meeting of the governors of this hospital was held in the board room of the new hospital in Holloway Road on Friday afternoon, 22nd ult. In view of the discussion on the pay ward question there was a large attendance of ladies and gentlemen. Mr. C. T. Murdoch, chairman of the hospital, presided. He was supported by Hon. Reginald Capel, hon. secretary, and Mr. Marshall Lang.

The annual report made special reference to the completion of the hospital buildings. The outlay upon the whole site and buildings since their inception was little short of £100,000; with the exception of £14,000 this amount had all been obtained. The number of in-patients during the year was 1,201, an increase of 110 over 1893, while the out-patients showed a slight decrease, viz., 25,083, against 26,390 in the previous year. The year ended with a deficiency of £1,561. The magnificent contributions to the funds of Mrs. Andrew Fernie (£1,100) and Rev. Francis Jacox (£1,050) were gratefully acknowledged. The report concluded with a reference to the pay wards which had been opened on October 1st. The payments are limited to £2 2s. per week, reducible in accordance with the means of the applicants, and at the discretion of the House Committee, there being no intention to make a profit.

The Chairman moved the adoption of the report. He congratulated the governors on their meeting for the first time in their new hospital. Their buildings were now completed. At present they could receive 103 patients, and they intended gradually to furnish other wards. The slight decrease in the number of out-patients was due to the more careful inquiries that had been instituted. (Hear, hear.) Their deficiency of £1,560 was not a very great sum in these days. It was worthy of note that henceforth there would be no commissions to their secretary. They had had to part with a portion of their reserve funds, but in realising they had made £1,152 of profits, which was very satisfactory. In establishing pay wards the committee felt that they had simply been carrying out the constitution of the hospital. (Hear, hear.) The governors might alter the constitution if they thought fit, but until then the committee had no power to depart from it. They had 19 pay beds in all, of which 12 were occupied. They were not, however, likely to forget that that was a general hospital, and their first duty was towards the free beds.

Mr. Marshall Lang having seconded, the report was adopted.

Votes of thanks were passed to the Committee of Management, to the medical staff, and to the members of the Ladies' Association, whose services were specially recognised. The

Committee of Management was re-elected, and Mr. Burdett-Coutts, M.P., and the Hon. Reginald Capel were re-elected hon. treasurer and hon. secretary respectively.

DR. GLOVER'S RESOLUTION.

Dr. GLOVER then moved the motion of which he had given notice. It was in the following terms:—

That this meeting of governors of the Great Northern Central Hospital regret the recent changes in the administration of the hospital, by which pay-patients have been admitted to its wards to be attended gratuitously by the honorary staff, and is of opinion that such a system is not likely to conduce to the welfare of the hospital or the advantage of the poor, for whom such institutions exist, and should without further delay be abandoned.

Dr. GLOVER hoped that no one would think that he had brought forward that motion in an unfriendly spirit. No subscription was given by him more cheerfully than his subscription to that hospital. He wished also to acknowledge the courtesy with which he had been assisted by the officials in investigating the whole subject. He would first submit that the system of pay wards that had been adopted in that hospital represented an entirely new departure, not only in regard to that hospital, but in regard to any general hospital in London. ("No; Guy's and St. Thomas's!") He denied that St. Thomas's formed a precedent. The paying patients there were attended not by the members of the honorary staff, but by the Resident Medical Officer who was paid. It was the same at Guy's. At the Great Northern Hospital paying patients were attracted by the eminence of their consulting staff. It was therefore a new departure, and he said deliberately it was not a good departure. Hospitals such as theirs existed for the poor—not, however, for the very poor who were provided for in the magnificent Metropolitan Asylums Board and Poor Law hospitals which cost the ratepayers about half a million in the year. As a governor he objected to his subscription being devoted to the assistance of those who could afford to pay for themselves. Before they attempted to cater for a new class of society he would ask if they had already provided adequately for the poor. As a matter of fact their hospital was inadequate for the needs of those for whom hospitals primarily existed. It was practically the only hospital in a parish with between 350,000 and 400,000 people. He would warn them that if the charitable public came to see that they were not going to attend to the needs of the poor, then subscriptions would certainly fall off. People in these days were so hardly pressed with rates and taxes that charities which were not most narrowly looked after would lose the support of the public. He cheerfully acknowledged that that hospital had set an example in the care with which people who were not proper objects of hospital relief were excluded. He doubted, however, whether they had yet wholly succeeded in keeping out those who had no right to be there. The chairman had said that they were bound by their constitution to have pay wards. (Hear, hear.) He thought that was scarcely accurate.

The CHAIRMAN read the first article of the constitution, which provides that the Great Northern Central Hospital "shall be a general and free hospital with pay wards."

Dr. GLOVER said there was nothing in that to justify the particular system of pay wards which had been adopted. (Hear, hear.) Their scheme had been adopted without notice having been given to the governors. ("No, no!") He had the highest authority for saying that the change had been introduced without notice. He (Dr. Glover) certainly never was consulted. What was the object of the change? Was it financial? ("No.") He was glad to hear that disclaimer. It was within his knowledge that there had been five cases of typhoid fever treated in the pay wards; why should they take in typhoid cases, which were costly and troublesome, and which were provided for in special institutions? One of the paying patients was from Canterbury. He did not see the necessity for admitting patients from Canterbury. Another patient was a carpenter earning 30s. a week whom they should all have been glad to receive into the free wards. ("No.") That movement in his opinion was calculated to exaggerate an evil tendency to resorting to hospitals. It was much better that patients who could at all be treated at home should be kept at home. He admitted that there were patients who could not be treated at home who could hardly be taken into the free wards. But for these ample provision was made in nursing homes. In that very district there were two or three such homes where patients could be accommodated on easy and most reasonable terms. In going into competition with the nurses, many of them thoroughly trained, they were going into a new business, and doing an injury to a worthy class of women. Why should you do so when so many of the poor for whom you exist to cater are unprovided with beds?

MR. THOMAS GRAHAM seconded. He could not reconcile himself to the idea of an hospital for the poor making money by pay wards. The system did injury to the medical practitioners in the district, whose fees were small in any case. He had been told that the pay system was of advantage to clerks and to men of that station, but he maintained that these had a higher claim upon the free wards than the poor themselves.

MR. BURDETT'S AMENDMENT.

The CHAIRMAN said that an amendment had been put in by Mr. Burdett which, no doubt, most of them had seen. He wished, however, to say that this amendment had not been sent out to the governors by authority of the Hospital Committee.

Mr. BURDETT said that the post-card containing the notice of the amendment had at the top of it his private address and the date. That being so, he was sure the Chairman would acquit him of any attempt to commit the committee. (Hear, hear.) His amendment was in the following terms:—

That with the two-fold object of loyally administering the affairs of the Great Northern Central Hospital on the lines of its constitution, and of reducing to a minimum the competition between the hospital and private practitioners, it is desirable that the system of pay wards now in operation be so modified that the patients in those wards may, if they desire it, be attended in the hospital by outside practitioners of their own selection; that the pay system, thus modified, be continued for an experimental period of five years; and that a committee of governors be appointed to prepare a detailed scheme on these lines, and to report to a meeting of the whole body of governors to be convened for this special purpose in June next.

Mr. G. W. SMYTHE rose to a point of order as to whether Mr. Burdett's motion could be moved without notice.

The CHAIRMAN ruled that as it was not a motion, but an amendment, Mr. Burdett was in order.

Mr. BURDETT, continuing, controverted Dr. Glover's statement that the pay system was a new departure. It was not a new departure at St. Thomas's, nor at Guy's, where patients were received into the general wards and treated by the members of the honorary staff at the rate of one guinea per week; nor was it a new departure in connection with that hospital. Constitutionally it would be quite unjustifiable to disassociate the pay system from that hospital. In the year 1882 he (Mr. Burdett) had addressed a meeting held in the Athenæum in Camden Road to discuss the question of an hospital for North London, and in his address on that occasion stress had been laid on the importance of having pay wards in the new hospital. At a large meeting held in Islington in 1883, when the Duke of Westminster presided, it was resolved to establish a hospital on the combined system of free and paying patients, and this was laid down in the constitution. As a matter of fact that hospital was the only hospital in the country that made full provision for free and paying patients. It had always been put forward that that was their system. As to this Mr. Burdett read an extract from the *Times* of the date when the hospital was established. Dr. Glover had said that their subscriptions would fall off. Their experience had been to the very contrary. That hospital had thriven during the recent times of depression more than any other in London. Since 1883 they had raised a sum of £55,000. North London had set a magnificent example to those in the West, and indeed to the whole country. Their invested funds had also gone up; they need have no fear of the falling off of subscriptions, for their income had doubled in ten years. It was said that if they gave up the pay wards, they would have the support of the medical staff, for then they would have 155 free beds, and be entitled to have a medical school. ("No.") He did not know as to that; but they did not want another medical school in London. All the tendencies were towards centralisation in medical education; moreover, with a medical school the expenses of maintaining the hospital would be much greater. Dr. Glover had said that patients who were able to pay had the nursing homes to go to. He (Mr. Burdett) had considerable experience in regard to nursing homes and home hospitals, and he knew that a nursing home which fulfilled every sanitary and hygienic requirement as that hospital did, could not afford to receive patients at the rates that this class of patient could pay. What the hospitals had to fight against was the pauperising tendencies of medical relief. As to the medical and general aspects of the question, he would remind Dr. Glover and the medical profession, as well as the public, that we have too much free and unrestricted medical relief in London at the present time. He had ascertained from the Registrar General, and the returns of the Hospital Sunday Fund, that the growth in the population and in the number of patients treated at the hospitals in London during the last two decades was as follows. From 1873 to 1883 the increase in the population and the increase in the number of patients relieved at the hospitals was identical, *i.e.*, 15½ per cent. In the decade 1883 to 1893 the population of London increased by only 10 per cent., but the number of patients treated at the hospitals increased by 48½ per cent. In twenty years ended 1893 the population increased by 28 per cent., whilst the number of patients increased by 75½ per cent. In other words, whereas in the ten years ended 1883 one out of every six of the population received hospital relief, in the decade ended 1893 one out of every four of the population was a hospital patient. Indiscriminate medical relief tended to pauperisation, and undermined the moral fibre of the people to an extent which could not be exaggerated. That was why he was so anxious that the Great Northern Central, the only hospital in London which had fully equipped pay wards, should keep up the system. But, with Dr. Glover, he objected to the form in which the pay system had been carried out. Their scheme did not provide for all the parties interested. For one thing, it did not provide for the general practitioners in the district. He (Mr. Burdett) supported a system by which a patient in the pay ward should be attended by his own medical man. That would enable the general practitioner in towns to have the same advantages of seeing hospital practice that the practitioner in the country had at the cottage hospital. Moreover, through the additional insight and experience obtained by the medical practitioner the whole district would benefit. He hoped that the time would come when they would be able to look within their own borders for members of their medical staff. He should like, for example, to see his friend, Dr. Glover, on the staff. There was no one more entitled to such an honour, and his ripe experience would be of immense advantage to the hospital. The amendment which he had submitted committed them to nothing until they had seen the actual scheme. He had not asked anyone to second it, as he wished the matter to be left in the hands of the governors entirely.

Dr. Potter seconded.

The Chairman said his colleagues and he could not look at that amendment in any other light than as a vote of censure.

Mr. Burdett protested that he had no intention of censuring the committee or anyone. He should be very glad that the Committee of Management should appoint the special committee referred to in the amendment.

The Chairman said that supposing the regulations were passed, for five years the governors would have no voice in making any alterations. (No.) How could they have outside medical men coming in for whom the hospital would be in no way responsible? Competition of that kind within the hospital was quite out of the question. The medical men in the district he was sure would not agree to it. He had put the amendment before Sir Francis de Winton, and, in his opinion, the illustrious personage who was the president of the hospital would object to it.

The amendment was then put to the meeting, but only the proposer and seconder voted for it, and it was rejected.

The Discussion.

Mr. Dewey supported Dr. Glover's motion. He grudged that accommodation which was already inadequate should be applied for a purpose which, in his opinion, notwithstanding that pay wards were mentioned in the constitution, had never been contemplated by those who had given the money for the hospital.

Mrs. Millman contended that the pay wards met a real need.

Mrs. Harkness asked why those who objected to pay wards had not protested while the wards were being put up? Great extra expense had been incurred by their erection, they would meet a felt want, and she must vote against Dr. Glover's resolution.

Dr. Stokes considered that the motion was ill-timed and badly worded. The agitation against the pay wards, which had been started by practitioners in the neighbourhood, would do the hospital harm. As a practitioner in the district, he (Dr. Stokes) was quite willing to put up with the infinitesimal martyrdom which the system involved.

Mr. Sawbridge, Mr. Kirby, and Mr. G. W. Smyth the representative of the Hospital Saturday Fund, who made an excellent little speech, having spoken, Dr. Glover's motion was put to the meeting, when 20 voted in favour and 26 against. The motion was accordingly rejected.

The meeting closed with a vote of thanks to the chairman.

